# PREVALENCES OF CARE PHENOMENA IN HOSPITAL AND LONG-TERM CARE – A CROSS-SECTIONAL STUDY

**DOI:** 10.1093/geroni/igaf122.2427

**Published:** 2025-12-31

**Authors:** Nico Haller, Simone Freitag, Tobias Melms, Steve Strupeit

**Affiliations:** University Medicine Greifswald, Greifswald, Mecklenburg-Vorpommern, Germany; University Medicine Greifswald, Greifswald, Mecklenburg-Vorpommern, Germany; University Medicine Greifswald, Greifswald, Mecklenburg-Vorpommern, Germany; University Medicine Greifswald, Greifswald, Mecklenburg-Vorpommern, Germany

## Abstract

Care dependency is a complex issue arising from physical, cognitive, or psychological conditions, collectively known as care phenomena. These phenomena can be significantly influenced by nursing interventions. While understanding the prevalence and identifying risk groups for care phenomena is crucial, there is a lack of up-to-date data in Germany. This study aims 1) to collect prevalence data on care phenomena in hospitals and long-term care settings, and 2) to investigate correlations between these care phenomena and the demographic variables age and gender. A cross-sectional study was conducted to investigate the care phenomena pain, falls, urinary and fecal incontinence, and pressure ulcers. Data collection was carried out in the setting hospital and long-term care facilities. The prevalence rates were calculated for each care phenomenon in the overall sample and for the two settings. Additionally, correlations between the care phenomena and with age- and gender were examined. In fall 2023, data was collected from 754 individuals. The results showed differences and similarities in prevalence data between long-term care and hospital settings. Similarities were observed for the prevalence of fall with 4.8%. Significant differences were found in the prevalence of urinary incontinence, with 26.6% in hospitals and 56.84% in long-term care. Moreover, significant correlations were identified between the care phenomena themselves, as well as with gender and age. This study provides current prevalence data on care phenomena in Germany. Future research should involve larger samples, in-depth analyses, and systematic investigations to enhance understanding and inform evidence-based practice, ultimately improving nursing care strategies.

